# The Burden of Dental Infections Among Hospitalized Patients With Rheumatoid Arthritis: A Cross-Sectional Analysis of the National Inpatient Sample Database

**DOI:** 10.7759/cureus.83568

**Published:** 2025-05-06

**Authors:** Gideon U Noah, Obinna V Ikwuka, Johncross C Nwadije, Memunat Y Ogunmefun, Ebube I Udeozor, Vivian Unachukwu, Hezborn M Magacha

**Affiliations:** 1 Internal Medicine, East Tennessee State University, Johnson City, USA; 2 Biostatistics and Epidemiology, East Tennessee State University, Johnson city, USA; 3 College of Dentistry, New York University, New York, USA; 4 Epidemiology and Biostatistics, East Tennessee State University, Johnson City, USA; 5 Public Health, Western Illinois University, Illinois, USA; 6 Internal Medicine, East Tennessee State University, Quillen College of Medicine, Johnson City, USA

**Keywords:** compromised immunity, dental infection, oral abscess, oral diseases, oral health care, oral health education, oral-systemic connection, race inequities, rheumatoid arthritis

## Abstract

Background

Oral infections such as cellulitis and abscesses of the oral cavity present significant public health burdens, particularly when they result in hospitalization. Rheumatoid arthritis (RA) is a systemic autoimmune condition that presents with chronic inflammation and has been linked to poor oral health outcomes. The immunological pathway that RA shares with periodontal diseases explains its impact on oral health outcomes. This study investigates the association between RA and oral infection-related hospitalizations among U.S. adults, utilizing data from the Healthcare Cost and Utilization Project’s (HCUP) National Inpatient Sample (NIS) from 2016 to 2022.

Methods

A cross-sectional, retrospective analysis was conducted on 14,975,196 adult inpatient records. This sample size was derived after filtering the total number of hospitalizations between 2016 and 2022 in the NIS dataset to include only adults (>18 years) with complete data for all the variables used in this analysis. The outcome variable was hospitalization for cellulitis or abscess of the mouth (CELL), identified using ICD-10-CM codes. The primary predictor was a diagnosis of RA. Covariates included age group, sex, race, length of hospital stays (LOS), smoking status (SMK), and the presence of systemic lupus erythematosus (SLE). Descriptive statistics, bivariate chi-square tests, and multivariate logistic regression were used to assess associations. Odds ratios (ORs) and 95% confidence intervals (CIs) were reported.

Results

Descriptive analysis indicated that only 10,710 (0.07%) of the total sample experienced oral infection-related hospitalizations. RA was present in 281,142 (1.88%) of patients. In bivariate analysis, RA was not significantly associated with CELL (p = 0.4306). However, when looking at multiple factors together, it was found that patients with RA were 40% more likely to be hospitalized for oral infections compared to those without RA. Other significant predictors included male sex (OR = 1.79), Black race (OR = 1.23), smoking (OR = 1.33), and longer LOS (OR = 1.44). Age was inversely related to oral infection risk, with patients aged 65 and older showing a 75% lower risk (OR = 0.25) compared to the 18-34-year-old reference group.

Conclusion

We found an independent link between RA and a higher likelihood of hospitalization for dental infections. This finding brings to light just how complex oral health disparities are driven not just by inflammation but also by factors like age, race, and health behaviors. These findings make a strong case for incorporating dental assessments into the routine care of RA patients and speak to the importance of focused prevention efforts. Future longitudinal research is recommended to establish causal relationships and to see how much of a difference approaches to collaborative care can make in cutting down hospitalizations related to oral infections.

## Introduction

Dental infections, particularly cellulitis and abscesses of the mouth, represent a serious yet often overlooked public health concern. Cellulitis is an infection in the fatty tissue that appears as swollen, painful, and red areas, and it is different from abscesses because it does not have pus [1]. These infections can escalate quickly, leading to facial swelling, systemic infection, airway compromise, and, in severe cases, hospitalization [2]. Besides the direct effects of untreated oral infections, more and more studies show that they are connected to systemic diseases, especially autoimmune and inflammatory conditions like rheumatoid arthritis (RA) [3].

RA is a chronic systemic autoimmune disease that primarily affects synovial joints but also has far-reaching impacts on other organ systems and the immune response [4]. Affecting approximately 1.3 million Americans, RA is associated with chronic systemic inflammation, immune dysregulation, and comorbid conditions, including cardiovascular and periodontal diseases [4]. The shared pathophysiological mechanisms between RA and periodontal conditions - such as elevated levels of pro-inflammatory cytokines (e.g., TNF-α, IL-6) and matrix metalloproteinases - suggest an immunoinflammatory connection where oral infections may be more common and more severe among individuals with RA [5, 6].

Given these immunological overlaps, it is biologically plausible that RA not only contributes to chronic periodontal deterioration but may also increase the likelihood of acute oral infections that require hospitalization, such as cellulitis or abscesses of the mouth. However, despite mounting evidence linking RA to oral health challenges, limited research has examined how RA influences inpatient outcomes specifically related to dental infections. Prior studies have largely focused on periodontal disease or outpatient oral health indices in RA populations, while inpatient metrics-such as infection-related admissions-remain understudied. Furthermore, the interplay between RA and social determinants of health - including age, race, sex, socioeconomic status, and modifiable behaviors like smoking - warrants deeper exploration to understand the broader systemic burden of oral disease in this vulnerable population [7, 8].

Hospital-based infections of dental origin pose clinical risks, but beyond that, they also have significant economic burdens. National estimates suggest that dental-related emergency department visits and subsequent admissions cost the U.S. healthcare system billions annually, with many cases deemed preventable through earlier intervention [9]. Patients with RA, already experiencing functional limitations, immunosuppression from medications, and greater healthcare utilization, may be at elevated risk for complications arising from oral infections that progress to systemic involvement.

This study uses information from the National Inpatient Sample (NIS), which is the biggest public database of all types of hospital care in the United States, to look at how dental infections-especially cellulitis and abscess in the mouth-affect hospitalized patients with RA. The objective of this study is twofold. Our first objective is to assess whether patients with RA have a significantly higher likelihood of being hospitalized for oral infections. Secondly, we wish to explore how demographic, behavioral, and clinical variables modify this relationship. By addressing these questions through a national, representative sample, our study provides critical insights into the interrelationship between oral and systemic health and aims to highlight potential gaps in preventive care and interdisciplinary healthcare delivery.

## Materials and methods

Study design and data source

This cross-sectional study utilized data from the NIS, part of the Healthcare Cost and Utilization Project (HCUP), for the years 2016 through 2022. These years were chosen to provide a contemporary and comprehensive understanding of inpatient trends while aligning with ICD-10-CM diagnostic coding implementation. Using this range also ensures sufficient statistical power to detect associations in relatively rare outcomes. The NIS is the largest publicly available all-payer inpatient database in the United States and provides weighted estimates representing approximately 35 million hospitalizations annually [10]. This dataset included de-identified information on patient demographics, diagnoses, procedures, hospital characteristics, and outcomes.

Study population

The inpatient study records we analyzed had a total of 14,975,196 patients. This sample included adult patients (≥18 years old) who were hospitalized during the study period (2016-2022) and met our inclusion criteria of having complete records for our selected variables (cellulitis and abscess of the mouth, RA, age, sex, race, LOS, SLE, smoking status, and hypertension smoking status). Records of patients under 18 years old had incomplete or missing data for the selected variables, and outpatient or non-hospitalized patients were excluded from this study. The primary outcome variable was hospitalization for a dental infection, specifically cellulitis and abscess of the mouth, identified using the ICD-10-CM code K12.2 [11]. The primary predictor variable was a diagnosis of RA, identified by ICD-10-CM codes M05 and M06 [11].

Covariates

Potential confounders and effect modifiers included age (divided into four groups: 18-34, 35-49, 50-64, and 65 years and older), sex (male or female), and race/ethnicity (grouped as White, Black, Hispanic, Asian/Pacific Islander, Native American, and Other/Unknown, where “Other/Unknown” comprised people from multiple races or those not listed above). Other factors considered were the length of hospital stay (LOS), which was split into four groups (0-7 days, 8-14 days, 15-21 days, and more than 21 days), smoking status (identified by ICD-10-CM code Z72.0) [11], and health issues like systemic lupus erythematosus (SLE) (identified using ICD-10-CM code M32.9) and uncomplicated hypertension (identified using clinical classification software categories). Other confounders included LOS categorized into four groups (0-7 days, 8-14 days, 15-21 days, >21 days) and a behavioral factor represented by smoking status (identified by ICD-10-CM code Z72.0) [11]. Clinical variables included SLE (identified using ICD-10-CM code M32.9) and uncomplicated hypertension (identified using clinical classification software categories).

Data management and cleaning

Data were imported and analyzed using SAS statistical software version 9.4 (SAS Inc., Cary, NC). Variables (age, sex, race, and LOS) were recoded for ease of interpretation, and missing data were handled through listwise deletion (Table 1). Records with missing values in any of the primary analysis variables were excluded to ensure consistency in the regression models.

**Table 1:** Variable Definitions, Original Coding, and Recoding Scheme

Variable Name	Variable Description	Original Response Options	Original Data Type	Recoding Plan	Recoded Response Options	Recoded Data Type
Age	Age of years of participants at the time of admission	0-124=Age in years	Ordinal	(.), (.A), (.B), (.C) will be recoded as missing.	1=18-34	Ordinal
		. =Missing			2=35-49	
		.A = Invalid			3= 50-64	
		.B = Unavailable from source			4= 65+	
		.C=Inconsistent				
Gender	Indicator of sex	0 = Male	Nominal	(.), (.A), (.C) will be recoded as missing.	0 = Male	Nominal
		1=Female			1 = Female	
		. = Missing				
		.A = Invalid				
		.C=Inconsistent				
Race	Race/ethnicity of patient	1 = White	Nominal	(.), (.A), (.B) will be recorded as missing.	1 = White	Nominal
		2 = Black			2 = Black	
		3 = Hispanic			3 =Hispanic	
		4 = Asian or Pacific Islander				
		5 = Native American			4 = Asian or Pacific Islander	
		6 = Other			5 = Native American	
		. = Missing				
		.A = Invalid			6 = Other/Unknown	
		.B = Unavailable from source				
Length of stay (LOS)	LOS in the hospital	0-365 = LOS in days	Ordinal	(.), (.A), (.B), (.C), will be recoded as missing.	1 = 0-7	Ordinal
		. = Missing				
		.A = Invalid			2 = 8-14	
		.B = Unavailable from source			3 = 15-21	
		.C = Inconsistent			4 = 21+	

Statistical analysis

Descriptive statistics were used to summarize demographic and clinical characteristics of the study population. A multivariate logistic regression model was then created to examine the independent association between RA and hospitalization for oral infections while controlling for covariates. Odds ratios (ORs) and 95% confidence intervals (CIs) were computed to quantify associations. A p-value <0.05 was considered statistically significant.

We weighted all analyses using discharge weights provided by HCUP to generate nationally representative estimates.

## Results

This study examined the adjusted association between RA and oral infection-related hospitalizations (CELL) using multivariate logistic regression. The final model included a total sample of 14,975,196 adult inpatients from the NIS (2016-2022).

Descriptive and bivariate analysis

This study analyzed a nationally representative sample of 14,975,196 hospitalized adults in the United States from the 2016-2022 NIS dataset. Among these, only 10,710 (0.07%) cases were hospitalized primarily for cellulitis or abscess of the mouth, highlighting the relative rarity but potential severity of dental infections requiring inpatient care (Table 2).

**Table 2:** Descriptive Statistics of Study Variables (n = 14,975,196) CELL: Cellulitis or abscess of the mouth; RA: rheumatoid arthritis; LOS: length of stay in days; SMK: smoking status; SLE: systemic lupus erythematosus

Variable	Category	Frequency (n)	Percentage (%)
CELL	Present	10,710	0.07
	Absent	14,964,486	99.93
RA	Present	281, 142	1.88
	Absent	14,694,054	98.12
Gender (Sex indicator)	Female	8,620,794	57.57
	Male	6,354,402	42.43
RACE	White	10,031,972	66.99
	Black	2,325,988	15.53
	Hispanics	1,669,442	11.15
	Asian/Pacific Islanders	405,055	2.70
	Native Americans	94,456	0.63
	Others/Unknown	448,283	2.99
AGE group (Years)	18-34	2,875,866	19.20
	35-49	2, 201,392	14.70
	50-64	3,566,951	23.82
	65 +	6,330,987	42.28
LOS	0-7	12,000,813	80.14
	8-14	2,131,355	14.23
	15-21	502,728	3.36
	21 +	340,300	2.27
SMK	Present	168, 360	1.12
	Absent	14,806,836	98.88
SLE	Present	76,709	0.51
	Absent	14,898,487	99.49

Approximately 281,142 (1.88%) of the cohort had a diagnosis of RA, while the remaining 14,964,486 (98.12%) did not. Females constituted a majority of the population, making up 8,620,794 (57.57%) of the study population, which aligns with known patterns of hospital utilization and RA prevalence.

Regarding race and ethnicity, White patients constituted 10,031,972 (66.99%) of the study population, making up the largest racial group. This was followed by 2,325,988 (15.53%) Black, 1,669,442 (11.15%) Hispanic, 405,055 (2.70%) Asian/Pacific Islander, and 94,456 (0.63%) Native American patients. Another 448,283 (2.99%) were categorized as “Other” or “Unknown,” indicating some variability or missingness in race reporting within the dataset (Table 2).

Age distribution showed that the majority of hospitalizations occurred in older adults, with 6,330,987 (42.28%) being patients aged 65 years or older, followed by patients aged 50-64 years making up 3,566,951 (23.82%); patients aged 35-49 years making up 2,201,392 (14.70%); and 2,875,866 (19.20%) aged 18-34 years. This age skew reflects the higher likelihood of hospitalization with advancing age and comorbidities.

For LOS, most patients, specifically 12,000,813 (80.14%), had short hospitalizations between 0 and 7 days. 2,131,355 (14.23%) patients stayed between 8 and 14 days, and a smaller number of patients, 843,028 (5.63%), required 15 or more days of hospitalization. This pattern is consistent with the typical clinical course for many acute medical conditions.

Smoking status was reported as present in 168,360 (1.12%) of patients, although underreporting is possible due to reliance on ICD coding. Similarly, SLE was rare in this population, present in only 76,709 (0.51%) of cases.

Going further, we used chi-square tests to evaluate the unadjusted associations between the covariates and the outcome variable (hospitalization for cellulitis or abscess of the mouth).

RA was not significantly associated with the outcome at the bivariate level (χ² = 0.62, p = 0.4306), suggesting that RA alone does not significantly predict hospitalization for oral infections (Table 3). However, RA is clinically relevant, and our findings caused us to include it in the multivariate model.

**Table 3:** Bivariate Associations Between Covariates and Outcome Variable (Cellulitis and abscess of the mouth)

Variable	Chi-Square (χ²)	p-Value	Significant Association?
Rheumatoid arthritis (RA)	0.62	0.4306	No
Female	524.02	<0.0001	Yes
Race	327.62	<0.0001	Yes
Age group	2937.38	<0.0001	Yes
Length of stay (LOS)	52.77	<0.0001	Yes
Smoking status (SMK)	64.49	<0.0001	Yes
Systemic lupus erythematosus (SLE)	2.28	0.1315	No

Sex showed a highly significant association (χ² = 524.02, p < 0.0001). Males were more likely than females to be hospitalized with oral infections, consistent with literature noting poorer oral health behaviors and outcomes among men.

Race was also significantly associated (χ² = 327.62, p < 0.0001), indicating that racial disparities exist in oral infection hospitalizations. This points to broader systemic inequities in oral healthcare access and preventive service utilization.

The bivariate analysis revealed a statistically significant association between age group and hospitalization for cellulitis and abscess of the mouth (χ² = 2937.38, p < 0.0001). Cross-tabulation showed that although the absolute number of infections was highest in older adults due to their population size, the proportion of individuals with oral infections relative to their age group size was highest among the youngest age groups. Specifically, patients aged 18-34 years (age group 1) had a high within-group infection rate, with 0.11% (3,097 out of 2,875,866) hospitalized for oral infections. This trend followed the 35-49 age group closely, which had a 0.13% (2,837 out of 2,201,392) infection rate. In contrast, those aged 65 and above had a markedly lower infection proportion of 0.03%.

LOS was significantly associated (χ² = 52.77, p < 0.0001), suggesting that patients with oral infections may have longer or more complex hospital courses.

Smoking status was also significantly linked to oral infections (χ² = 64.49, p < 0.0001), aligning with the known negative impact of smoking on oral tissue health and immune function.

SLE, on the other hand, was not significantly associated with oral infection hospitalizations (χ² = 2.28, p = 0.1315), though it remains a clinically relevant condition due to its immunosuppressive features (Table 3).

Multivariate analysis

After adjusting for sex, race, age group, LOS, smoking status, and SLE, the association between RA and oral infections became statistically significant.

Patients with RA had 1.40 times the odds of having oral infection-related hospitalizations compared to those without RA (OR = 1.399, 95% CI: 1.210-1.618, p < 0.0001). Other significant predictors included male sex (OR = 1.79), Black race (OR = 1.23), smoking (OR = 1.33), and longer hospital stays (OR = 1.44). Increasing age was associated with significantly lower odds of oral infection, with patients aged 65+ having the lowest risk (OR = 0.25) (Table 4).

**Table 4:** Adjusted Odds Ratios for Predictors of Cellulitis and Abscess of the Mouth RA: rheumatoid arthritis; Ref: reference; LOS: length of stay; SMK: smoking status; SLE: systemic lupus erythematosus

Predictor Variable	Values	Odds Ratio	95% Confidence Interval	p-value
Rheumatoid Arthritis (RA)	Absent	Ref	Ref	Ref
	Present	1.399	1.210–1.618	<0.0001
Female	Male	Ref	Ref	Ref
	Female	0.558	0.536–0.581	<0.0001
Race	White	Ref	Ref	Ref
	Asian/Pacific Islanders	0.699	0.609–0.802	<0.0001
	Black	1.227	1.170–1.287	<0.0001
	Hispanic	0.846	0.795–0.901	<0.0001
	Native Americans	0.941	0.751–1.180	0.5996
	Others	0.866	0.772–0.971	0.0136
Age	18-34	Ref	Ref	Ref
	35-49	1.034	0.982–1.089	0.2076
	50-64	0.566	0.536–0.597	<0.0001
	65 +	0.248	0.234–0.263	<0.0001
LOS	0-7	Ref	Ref	Ref
	8-14	1.104	1.044–1.167	0.0005
	15-20	1.156	1.042–1.282	0.0062
	21 +	1.436	1.291–1.598	<0.0001
SMK	Absent	Ref	Ref	Ref
	Present	1.330	1.159–1.526	<0.0001
SLE	Absent	Ref	Ref	Ref
	Present	1.185	0.928–1.513	0.1727

**Figure 1:**
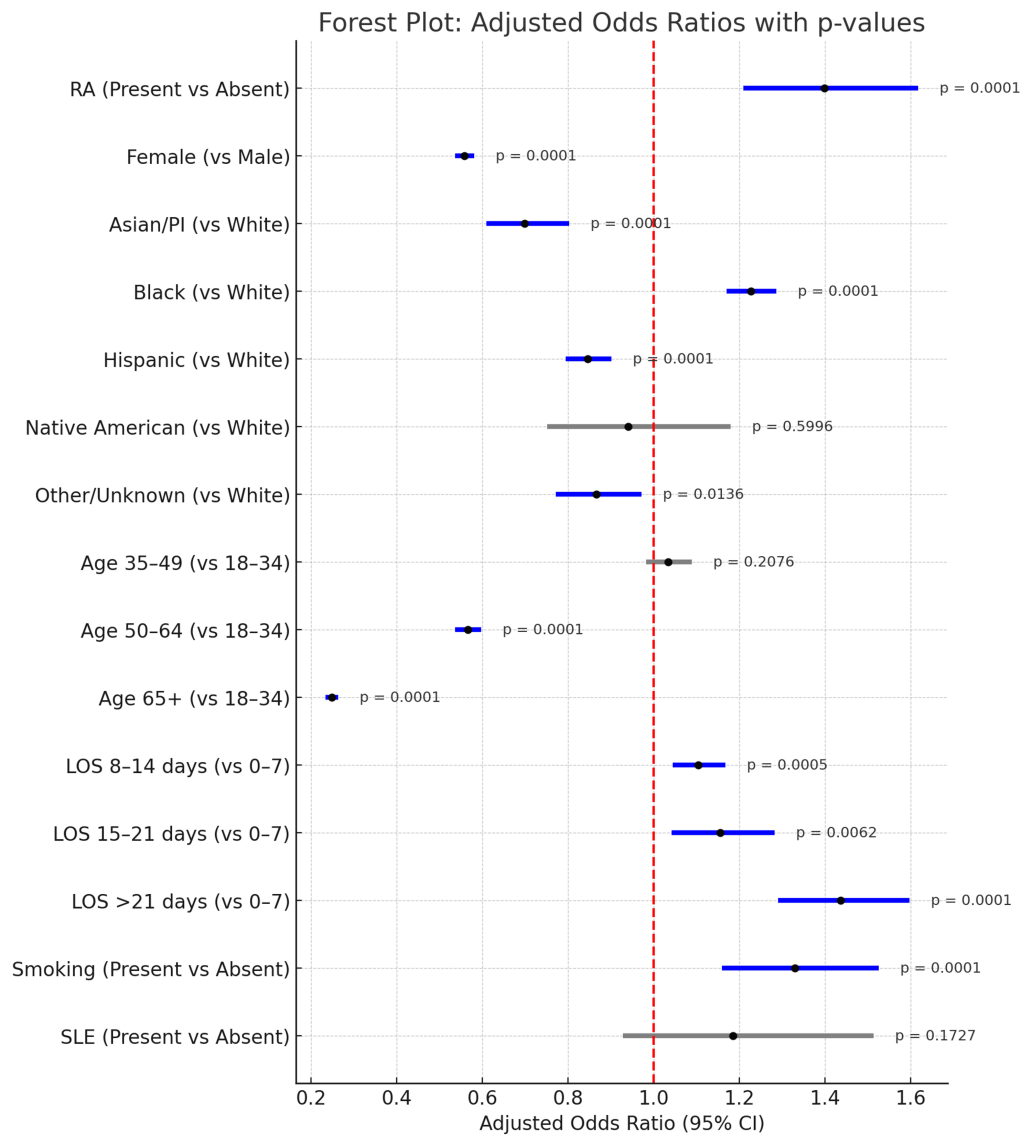
Forest Plot of Predictors of Oral Infection Hospitalization Significant predictors are shown in blue, while non-significant predictors are in gray. LOS: length of stay; SLE: systemic lupus erythematosus

We constructed a forest plot (Figure 1) to visually summarize the adjusted ORs and their CIs for all predictor variables. The plot clearly illustrates which factors were significantly associated with oral infection hospitalizations, with variables having p-values <0.05 being regarded as significant. Significant predictors are shown in blue, while non-significant ones are in gray. It also displays the adjusted ORs, 95% CIs, and corresponding p-values for each variable.

These findings demonstrate a significant and independent relationship between RA and oral infection hospitalizations. They also emphasize the relevance of sociodemographic, behavioral, and clinical factors in understanding oral-systemic disease interactions in hospitalized adults.

## Discussion

This study highlights the prevalence of dental infections among hospitalized patients with RA, offering new insight into the relationship between oral health and systemic inflammatory illnesses. RA contributes to poor oral health outcomes, including severe conditions such as cellulitis and abscess of the mouth, through a complex interplay of systemic inflammation, immune dysregulation, and microbial susceptibility. Central to RA’s pathology is the persistent activation of pro-inflammatory cytokines such as tumor necrosis factor-alpha (TNF-α), interleukin-6 (IL-6), and interleukin-1 beta (IL-1β), which not only perpetuate joint destruction but also compromise mucosal immunity and tissue integrity in the oral cavity [12]. These cytokines promote the recruitment of neutrophils and monocytes into periodontal and periapical tissues, exacerbating local inflammation and increasing the risk of deep-seated infections [13]. Additionally, RA is linked to an imbalance in the oral bacteria, especially with more Porphyromonas gingivalis, a pathogen that is involved in both periodontitis and autoimmune disorders because it can change host proteins and trigger autoimmunity [14]. The treatments often used for RA, like corticosteroids and disease-modifying antirheumatic drugs (DMARDs) such as methotrexate, can weaken the body's defenses and make patients more likely to develop oral opportunistic infections [15]. Collectively, these mechanisms provide a plausible biological explanation for the increased risk of severe oral infections in individuals with RA.

Our analysis of over 14 million hospitalizations in the United States between 2016 and 2022 reveals a statistically significant association between RA and increased odds of hospitalization due to cellulitis and abscess of the mouth. After controlling for demographic, behavioral, and clinical confounders, patients with RA had a 40% higher likelihood of presenting with these dental infections. This result adds to and validates earlier research highlighting RA patients’ increased susceptibility to infections as a result of immunosuppressive medications and immunological dysregulation [6, 16-17].

The significance of this relationship is underscored by the observed associations between other variables and oral infection risk. Notably, females were significantly less likely than males to be hospitalized for oral infections, a trend previously observed in other oral health surveillance studies [8]. Racial disparities also emerged, with Black patients showing higher odds of dental infection-related hospitalizations compared to White patients, while Hispanic and Asian patients had lower odds. These disparities are reflective of systemic inequities in healthcare access, oral health literacy, and socioeconomic status, which have been well-documented in literature [18, 19].

Age and LOS were strong predictors of dental infection risk. Interestingly, our study revealed that younger adults, particularly those aged 18-34 and 35-49, had the highest within-group rates of hospitalization for oral infections. Specifically, 0.11% of individuals aged 18-34 and 0.13% of those aged 35-49 were hospitalized for cellulitis or abscess of the mouth. Although older age groups had higher absolute numbers of hospitalizations, the infection rates relative to population size were notably higher in these younger cohorts. This finding is unexpected, as younger adults are generally considered to be at lower risk for serious infections [20]. However, it may reflect barriers to preventive dental care, including lack of insurance coverage, reduced healthcare-seeking behavior, or limited oral health literacy in this age group. Previous research has documented that young adults are among the least likely to utilize routine dental services, often waiting until symptoms become severe before seeking care [21]. These patterns highlight a critical opportunity for targeted public health interventions aimed at improving early access to dental care and education among younger populations, potentially preventing progression to more severe infections that require hospitalization [9]. In contrast, patients with longer hospital stays (LOS >20 days) were significantly more likely to have been hospitalized for oral infections, possibly indicating more severe infections or comorbid complications that necessitated extended care. For example, a study by James et al. (2025) showed that delays in receiving timely surgical treatment for dental infections led to longer hospital stays and more changes in antibiotic treatment, highlighting how important it is to get treatment on time to lessen the strain on hospitals [22].

Behavioral risk factors, such as smoking, were also positively associated with dental infection risk. This aligns with research demonstrating the immunosuppressive and tissue-destructive effects of smoking on oral health [7]. At the same time, SLE did not show a strong enough link in our study, indicating that even though SLE is another autoimmune disease, its connection to dental infections might be different from RA and needs more research.

The implications of these findings are multifaceted. From a clinical perspective, healthcare providers should consider integrating routine dental assessments and preventive care referrals into the management plans of RA patients. Interdisciplinary care, which bridges the fields of dentistry and rheumatology, may help prevent severe oral infections and their systemic effects, especially in light of the growing evidence that links systemic inflammation and periodontal health [6].

From a public health approach, our findings advocate for increased access to dental care, particularly for people with chronic inflammatory disorders. Community-level interventions aimed at oral health education, routine screenings, and low-cost treatment for at-risk groups may reduce the subsequent load on inpatient healthcare systems. For example, rheumatology clinics might incorporate oral health examinations into routine visits and collaborate with dental providers to address oral health issues as soon as possible. Early detection and treatment of oral infections can save hospitalizations. Second, we can monitor the oral health of immunocompromised groups more closely by working with the CDC and using tools like the Behavioral Risk Factor Surveillance System (BRFSS) or by creating specific oral health programs for these groups. These data-driven approaches can identify regional disparities and guide interventions. We should push for policy recommendations to include oral health assessment and referral pathways in clinical practice guidelines for RA management. Guidelines by bodies like the American College of Rheumatology could incorporate dental health metrics. This will help to institutionalize the value of oral-systemic care within chronic disease frameworks.

Strengths and limitations

One of the major strengths of our study is our use of the NIS, which provided an extensive dataset that enhances the generalizability of the findings to the U.S. adult population. The use of weighted estimates ensures representation across a wide demographic and geographic range. Future researchers can replicate or expand on this study by incorporating additional years or comparing trends over time. Another strength is the size of our study population. Having analyzed 14 million hospitalizations, the study has strong statistical power to detect even modest associations between RA and oral infections. What this finding does for subsequent studies is that it offers a scalable model for future population-based studies across diverse clinical settings. Furthermore, the focus on oral-systemic interactions-especially the underexplored link between RA and severe dental infections-fills a critical gap in both the dental and medical literature. The result sets the stage for interdisciplinary studies involving rheumatologists, dentists, and public health professionals, paving the way for interdisciplinary and longitudinal research. Furthermore, the large sample size and robust statistical power enable researchers to explore subtler associations or stratify by subgroups.

Our study is not void of limitations. Our study couldn’t establish causality due to its observational and cross-sectional nature. Prospective longitudinal studies are needed to confirm temporal relationships and determine causal pathways. Secondly, the reliance on ICD-10 codes may lead to misclassification bias if conditions are under- or over-coded. For future studies, validation studies using chart review or clinical data can help assess the accuracy of administrative coding. Finally, our study only included hospitalized patients, potentially underestimating the full burden of dental infections in the RA population. To cater for this limitation in subsequent studies, including emergency department or outpatient claims data would present a more comprehensive picture of oral health service utilization.

To determine whether improved outpatient oral healthcare access can lower hospital admissions among immunocompromised populations, more research is required.

## Conclusions

In conclusion, this study provides compelling evidence that patients with RA are significantly more likely to be hospitalized for dental infections, such as cellulitis and abscess of the mouth. Our findings support the growing consensus that oral diseases do not occur in isolation but rather intersect with broader health trajectories, particularly in immunocompromised populations.

Going forward, these insights should inform multidisciplinary models of care that integrate dental services into chronic disease management frameworks, especially for autoimmune conditions. Moreover, public health initiatives should prioritize early dental screenings, targeted education campaigns, and accessible oral healthcare for high-risk groups. Through such systemic efforts, the burden of preventable oral infections - and their downstream complications - may be significantly reduced.

## References

[1] de-Vicente-Rodríguez JC (2004). Maxillofacial cellulitis. (Article in Spanish). Med Oral Patol Oral Cir Bucal.

[2] Robertson D, Smith AJ (2009). The microbiology of the acute dental abscess. J Med Microbiol.

[3] Jung ES, Choi YY, Lee KH (2019). Relationship between rheumatoid arthritis and periodontal disease in Korean adults: Data from the Sixth Korea National Health and Nutrition Examination Survey, 2013 to 2015. J Periodontol.

[4] Smolen JS, Aletaha D, McInnes IB (2016). Rheumatoid arthritis. Lancet.

[5] Eriksson K, Nise L, Kats A (2016). Prevalence of periodontitis in patients with established rheumatoid arthritis: A Swedish population based case-control study. PLoS One.

[6] Kaur S, White S, Bartold PM (2013). Periodontal disease and rheumatoid arthritis: A systematic review. J Dent Res.

[7] Cecoro G, Annunziata M, Iuorio MT, Nastri L, Guida L (2020). Periodontitis, low-grade inflammation and systemic health: A scoping review. Medicina (Kaunas).

[8] Peres MA, Macpherson LMD, Weyant RJ (201920). Oral diseases: A global public health challenge. Lancet.

[9] Owens PL, Manski RJ, Weiss AJ (2021). “Emergency Department Visits Involving Dental Conditions, 2018”. Healthcare Cost and Utilization Project (HCUP) Statistical Briefs [Internet].

[10] (2024). Agency for Healthcare Research and Quality. (n.d.). Overview of the National (Nationwide) Inpatient Sample (NIS). Healthcare Cost and Utilization Project. https://hcup-us.ahrq.gov/nisoverview.jsp?.

[11] (2025). Centers for Disease Control and Prevention, & National Center for Health Statistics. (2024). International classification of diseases, 10th revision, clinical modification (ICD-10-CM). https://icd10cmtool.cdc.gov/?fy=FY2024.

[12] McInnes IB, Schett G (2011). The pathogenesis of rheumatoid arthritis. N Engl J Med.

[13] Bartold PM, Marshall RI, Haynes DR (2005). Periodontitis and rheumatoid arthritis: A review. J Periodontol.

[14] Wegner N, Wait R, Sroka A (2010). Peptidylarginine deiminase from Porphyromonas gingivalis citrullinates human fibrinogen and α-enolase: implications for autoimmunity in rheumatoid arthritis. Arthritis Rheum.

[15] Blaess J, Walther J, Petitdemange A, Gottenberg JE, Sibilia J, Arnaud L, Felten R (2020). Immunosuppressive agents for rheumatoid arthritis: a systematic review of clinical trials and their current development stage. Ther Adv Musculoskelet Dis.

[16] Winthrop KL (2006). Serious infections with antirheumatic therapy: Are biologicals worse?. Ann Rheum Dis.

[17] Listing J, Strangfeld A, Kary S (2005). Infections in patients with rheumatoid arthritis treated with biologic agents. Arthritis Rheum.

[18] Dye BA, Li X, Beltran-Aguilar ED (2012). Selected oral health indicators in the United States, 2005-2008. NCHS Data Brief.

[19] Jackson SL, Vann WF Jr, Kotch JB, Pahel BT, Lee JY (2011). Impact of poor oral health on children's school attendance and performance. Am J Public Health.

[20] Esme M, Topeli A, Yavuz BB, Akova M (2019). Infections in the elderly critically-ill patients. Front Med (Lausanne).

[21] Nasseh K, Vujicic M (2014). The effect of growing income disparities on U.S. adults' dental care utilization. J Am Dent Assoc.

[22] N James J, Bloomquist R, Brown K, Looney S, Walker D, Day T (2025). Associations of time to the operating room on outcomes in odontogenic infection. BMC Oral Health.

